# Supplementary material for: Accurate Multiplex qPCR Detection of Epstein–Barr Virus/Cytomegalovirus/BK Virus in Kidney Transplant Patients: Pilot Study
Source: Int J Mol Sci. 2024 Nov 26;25(23):12698. doi: 10.3390/ijms252312698 (PMC11641394; doi:10.3390/ijms252312698)
Supplement: Supplementary file 1 [file ijms-25-12698-s001.zip › ijms-3294186-supplementary.pdf]

No.	Sex	Age	CKD diagnosis	CKD stage	ADPKD	Donor	Previous rejection events	Corticoid-sensitive rejection	Chronic graft rejection	Tacrolimus dose (mg)	Sirolimus dose (mg)	Mycophenolate dose (mg)	Cyclosporine dose (mg)	Prednisone dose (mg)	Valganciclovir dose (mg)	Rituximab dose (mg)	Adverse effects	WBC	RBC	HGB	Serum Creatinine	Serum Urea	Blood Glucose	eGFR	Blood Tacrolimus Level	Cyclosporine A C0	Cyclosporine A C2	Hematuria	Leukocyturia
**EBV - positive**																													
12	F	48	2011	G4A1	No	Cadaver	1	Yes	Yes	3	1			5			Digestive+leukopenia	4.89	4.75	12.6	3.08	66	83	17.09	6.3			NEG	NEG
52	M	73	2004	2	No	Live	0	-	No	5		2000						6.54	4.83	13.9	1.15	52	91	62.8	8.6			NEG	NEG
57	M	35	2018	T1	No	Cadaver	0	-	No	6		1440		17.5	900			9.05	3.98	13.2	1.36	29	139	66.94				POS	POS
93	M	22	2020	-	No	Cadaver	1	Yes	No	4		1440		5			Digestive	6.85	4.65	14.3	1.03	50	122	102.65	8.7			NEG	POS
67	M	70	2014	G2A1	No	Cadaver	0	-	No	4.5		1440		5				9.4	4.87	14.1	1.98	88	135	33.26	7			NEG	NEG
**CMV - positive**																													
No.	Sex	Age	CKD diagnosis	CKD stage	ADPKD	Donor	Previous rejection events	Corticoid-sensitive rejection	Chronic graft rejection	Tacrolimus dose (mg)	Sirolimus dose (mg)	Mycophenolate dose (mg)	Cyclosporine dose (mg)	Prednisone dose (mg)	Valganciclovir dose (mg)	Rituximab dose (mg)	Adverse effects	WBC	RBC	HGB	Serum Creatinine	Serum Urea	Blood Glucose	eGFR	Blood Tacrolimus Level	Cyclosporine A C0	Cyclosporine A C2	Hematuria	Leukocyturia
14	F	44	2018	G3b	No	Cadaver	1	No	No	1.5		1080		5		500	Digestive	10.25	4.26	12.3	2.11	99	98	27.75				NEG	NEG
24	M	37	2003	G3aA1	No	Live + Cadaver	1	Yes	Yes	4		1440		5				7.7	4.33	13.5	1.36	53	90	65.99	11.9			NEG	POS
34	M	51		T3	No	Cadaver	0		No	0		1440	200	5	450			9.92	4.12	12.9	2.04	68	105	36.65		145.7	639.6	POS	POS
68	M	45	2014		No	Cadaver	0		Yes	0		1440	100					9.6	4.06	11.5	1.92	70	404	41.13		103.6	659.7	NEG	NEG
77	F	52	1997	G3bA1	No	Live	1	Yes	No	2.5		1000		5				4.04	3.09	9.5	1.79	95	107	32.01	4.9			NEG	NEG
79	M	58	1998		No	Cadaver	0		No	3		1080			900			6.72	4.29	12.1	1.7	77	91	43.46				POS	POS
85	M	44	2002	G1A1	No	Live	0		No	0		1440	150	5				6.19	4.17	12.1	1.42	46	87	59.63	4		628	NEG	NEG
116	M	54	2004	G4A1	No	Live	3	Yes	Yes	3.5		1080		5				6.03	3.39	10.4	3.58	115	99	18.17	4.1				
**BKV - positive**																													
No.	Sex	Age	CKD diagnosis	CKD stage	ADPKD	Donor	Previous rejection events	Corticoid-sensitive rejection	Chronic graft rejection	Tacrolimus dose (mg)	Sirolimus dose (mg)	Mycophenolate dose (mg)	Cyclosporine dose (mg)	Prednisone dose (mg)	Valganciclovir dose (mg)	Rituximab dose (mg)	Adverse effects	WBC	RBC	HGB	Serum Creatinine	Serum Urea	Blood Glucose	eGFR	Blood Tacrolimus Level	Cyclosporine A C0	Cyclosporine A C2	Hematuria	Leukocyturia
16	M	42	2008	G2	No	Live + Cadaver	2	Yes	Yes	4		1440		20	900			13.25	3.8	12.9	1.91	65	89	42.29				NEG	NEG
25	M	54	2006	4	Yes	Cadaver	0	0	No	1		1440		5				11.03	3.93	11.6	2.32	117	95	30.7	4.9			POS	NEG
32	M	40	1998	G3aA1	No	Cadaver	0	0	No	11		1440		5				10.35	5.05	15.4	1.62	51	115	52.3	7.8			NEG	NEG
39	M	69		G2A1	No		0	0	No	3		360		5				5.99	5.29	16.6	1.01	49	155	75.55	9.6			NEG	NEG
41	M	64	2003	G2A1	No	Live	1	No	Yes	6		2000						8.19	4.65	14.6	1.32	50	99	56.62	9.7			NEG	NEG
82	F	47	2017	5D	Yes	Cadaver	0	0	No	2		1440		5				8.96	4.6	10.1	0.89	48	93	77.2	11.6			NEG	POS
103	M	49	2017	G2	No	Cadaver	0	0	No	2		720		5			Digestive + leukopenia	6.87	4.67	11.2	1.04	32	90	83.94	9.8			POS	POS
104	M	30	2022	T1	No	Cadaver	0	0	No	8		1080		5	450			3.6	4.62	14.8	1.08	24	91	91.62	14.5			POS	NEG
135	M	44	2019	G4	Yes	Cadaver	0	0	No	3.5		360		5				5.32	4.79	14.3	1.97	76		40.14	9.2			NEG	NEG

Figure S1 - Laboratory parameters of the positive patients.

CKD - chronic kidney disease, ADPKD - autosomal dominant polycystic kidney disease, Adverse effects - related to the immunosupressive medication, WBC - white blood cell count x10^3/µL, RBC - red blood cell count x10^6/µL, HGB - haemoglobin, Serum creatinine mg/dL, Serum urea mg/dL, Blood glucose g/dL, eGFR mL/min/1,73sqm, Blood Tacrolimus Level ng/mL, Cyclosporine A C0 ng/mL, Cyclosporine A C2 ng/mL.

Supplementary material

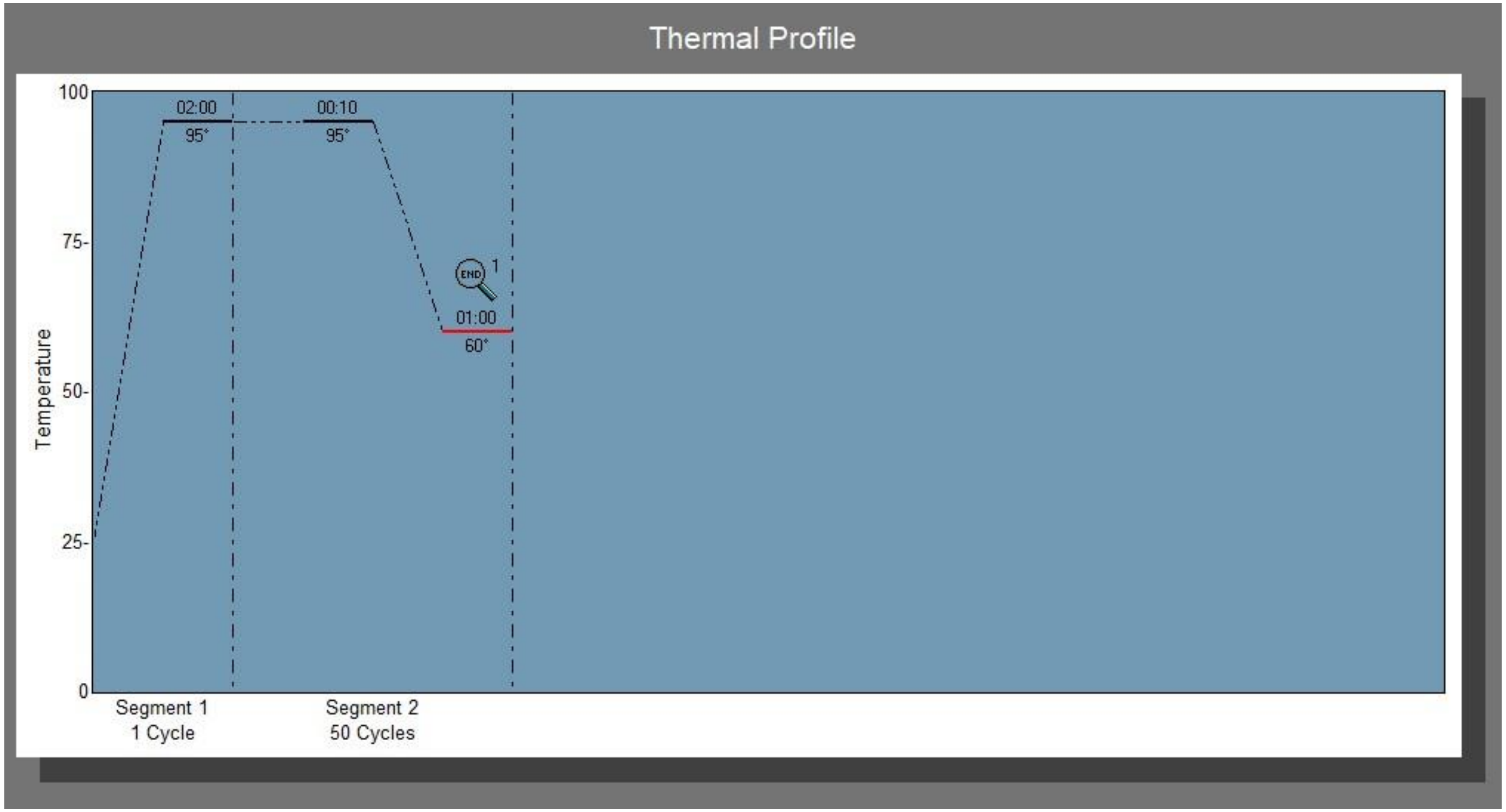

Figure S2: The thermal profile of the experiment.

All	1	2	3	4	5	6	7	8	9	10	11	12
A												
B		Standard<br>2.00e+005<br>2.00e+005<br>2.00e+005<br>2.00e+005	Standard<br>2.00e+004<br>2.00e+004<br>2.00e+004<br>2.00e+004	Standard<br>2.00e+003<br>2.00e+003<br>2.00e+003<br>2.00e+003	Standard<br>2.00e+002<br>2.00e+002<br>2.00e+002<br>2.00e+002	Standard<br>2.00e+001<br>2.00e+001<br>2.00e+001<br>2.00e+001	Standard<br>2.00e+000<br>2.00e+000<br>2.00e+000<br>2.00e+000					
C												
D		Unknown<br>CY5<br>ROX<br>HEX<br>FAM<br>1	Unknown<br>CY5<br>ROX<br>HEX<br>FAM<br>2	Unknown<br>CY5<br>ROX<br>HEX<br>FAM<br>3	Unknown<br>CY5<br>ROX<br>HEX<br>FAM<br>4	Unknown<br>CY5<br>ROX<br>HEX<br>FAM<br>5	Unknown<br>CY5<br>ROX<br>HEX<br>FAM<br>6	Unknown<br>CY5<br>ROX<br>HEX<br>FAM<br>7	Unknown<br>CY5<br>ROX<br>HEX<br>FAM<br>8	Unknown<br>CY5<br>ROX<br>HEX<br>FAM<br>9	Unknown<br>CY5<br>ROX<br>HEX<br>FAM<br>10	
E		Unknown<br>CY5<br>ROX<br>HEX<br>FAM<br>11	Unknown<br>CY5<br>ROX<br>HEX<br>FAM<br>12	Unknown<br>CY5<br>ROX<br>HEX<br>FAM<br>13	Unknown<br>CY5<br>ROX<br>HEX<br>FAM<br>14	Unknown<br>CY5<br>ROX<br>HEX<br>FAM<br>15	Unknown<br>CY5<br>ROX<br>HEX<br>FAM<br>16	Unknown<br>CY5<br>ROX<br>HEX<br>FAM<br>17	Unknown<br>CY5<br>ROX<br>HEX<br>FAM<br>18	Unknown<br>CY5<br>ROX<br>HEX<br>FAM<br>19	Unknown<br>CY5<br>ROX<br>HEX<br>FAM<br>20	
F		Unknown<br>CY5<br>ROX<br>HEX<br>FAM<br>21	Unknown<br>CY5<br>ROX<br>HEX<br>FAM<br>22	Unknown<br>CY5<br>ROX<br>HEX<br>FAM<br>23	Unknown<br>CY5<br>ROX<br>HEX<br>FAM<br>24	Unknown<br>CY5<br>ROX<br>HEX<br>FAM<br>25	Unknown<br>CY5<br>ROX<br>HEX<br>FAM<br>26	Unknown<br>CY5<br>ROX<br>HEX<br>FAM<br>27	Unknown<br>CY5<br>ROX<br>HEX<br>FAM<br>28	Unknown<br>CY5<br>ROX<br>HEX<br>FAM<br>29	Unknown<br>CY5<br>ROX<br>HEX<br>FAM<br>30	
G												
H											NTC<br>CY5<br>ROX<br>HEX<br>FAM	NTC<br>CY5<br>ROX<br>HEX<br>FAM

Figure S3: Plate set-up for session I. B 2 - 7: positive control dilutions; H11, H12: negative controls; D 2 - 11, E2 - 11, G 2 - 11: DNA samples tested for EBV/CMV/BKV (samples 1 - 30); D 2 - 11, E2 - 11, G 2 - 11: internal extraction control.

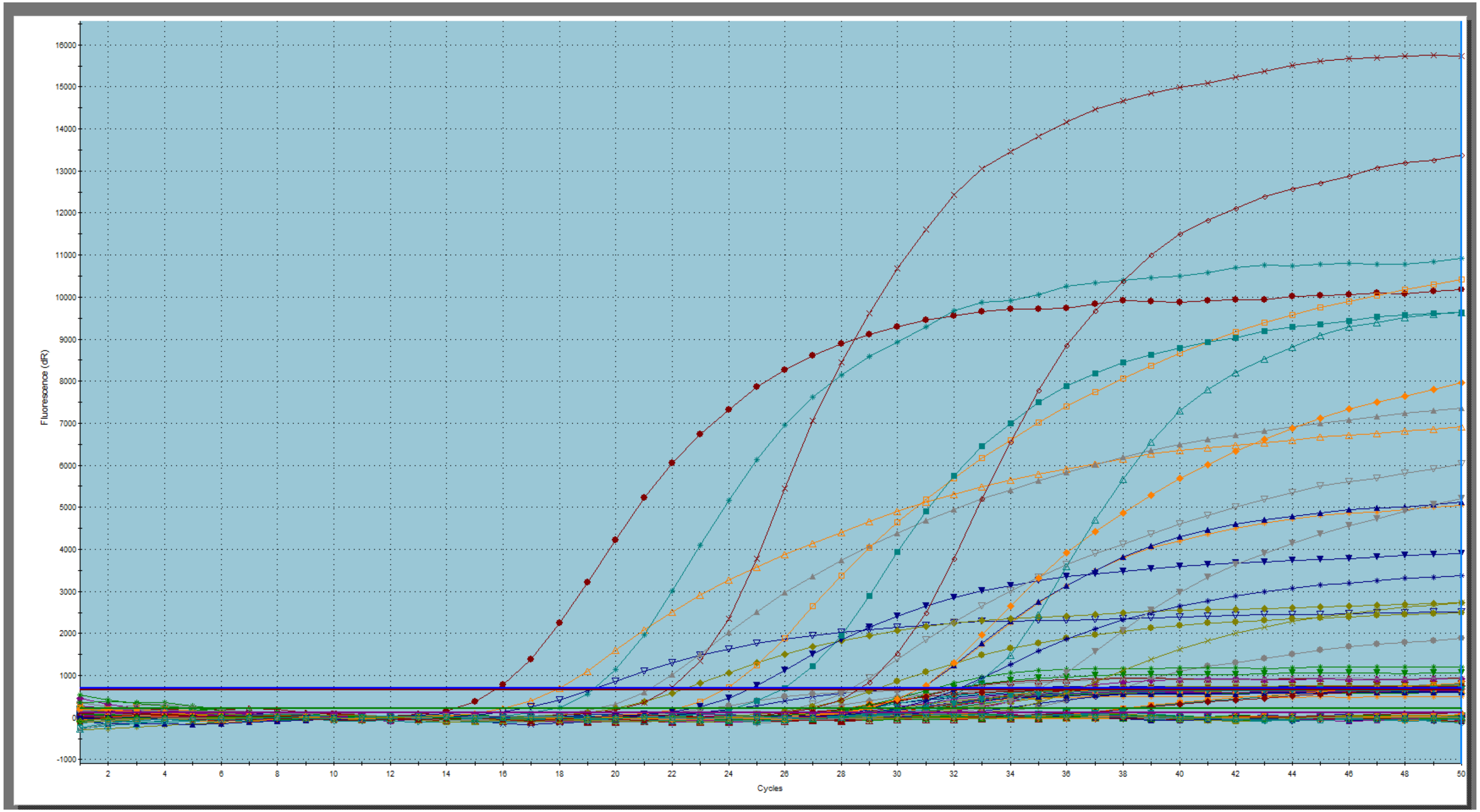

Figure S4: The amplification curves for all samples in the plate. Each line represents the amplification plot of wells B2-B7 (standards) and D2-D11, E2-E11, and F2-F11 (patient samples).

All	1	2	3	4	5	6	7	8	9	10	11	12
A												
B		Standard<br>No Ct<br>17.92<br>16.78<br>15.84	Standard<br>No Ct<br>21.20<br>20.09<br>19.31	Standard<br>No Ct<br>23.87<br>22.81<br>21.93	Standard<br>No Ct<br>28.33<br>26.80<br>25.98	Standard<br>No Ct<br>30.77<br>30.03<br>28.73	Standard<br>No Ct<br>34.98<br>34.17<br>32.47					
C												
D		Unknown<br>27.38<br>No Ct<br>No Ct<br>No Ct<br>(1)	Unknown<br>29.97<br>No Ct<br>No Ct<br>No Ct<br>(2)	Unknown<br>29.53<br>No Ct<br>No Ct<br>No Ct<br>(3)	Unknown<br>25.74<br>No Ct<br>No Ct<br>No Ct<br>(4)	Unknown<br>28.59<br>No Ct<br>No Ct<br>No Ct<br>(5)	Unknown<br>27.15<br>No Ct<br>No Ct<br>No Ct<br>(6)	Unknown<br>28.41<br>No Ct<br>No Ct<br>No Ct<br>(7)	Unknown<br>29.77<br>No Ct<br>No Ct<br>No Ct<br>(8)	Unknown<br>22.94<br>No Ct<br>No Ct<br>No Ct<br>(9)	Unknown<br>27.35<br>No Ct<br>No Ct<br>No Ct<br>(10)	
E		Unknown<br>29.86<br>No Ct<br>No Ct<br>No Ct<br>(11)	Unknown<br>21.91<br>No Ct<br>No Ct<br>38.00<br>(12)	Unknown<br>31.15<br>No Ct<br>No Ct<br>No Ct<br>(13)	Unknown<br>27.58<br>No Ct<br>38.43<br>No Ct<br>(14)	Unknown<br>31.52<br>No Ct<br>No Ct<br>No Ct<br>(15)	Unknown<br>29.18<br>30.77<br>No Ct<br>No Ct<br>(16)	Unknown<br>32.19<br>No Ct<br>No Ct<br>No Ct<br>(17)	Unknown<br>29.32<br>No Ct<br>No Ct<br>No Ct<br>(18)	Unknown<br>27.30<br>No Ct<br>No Ct<br>No Ct<br>(19)	Unknown<br>27.24<br>No Ct<br>No Ct<br>No Ct<br>(20)	
F		Unknown<br>30.73<br>No Ct<br>No Ct<br>No Ct<br>(21)	Unknown<br>30.21<br>No Ct<br>No Ct<br>No Ct<br>(22)	Unknown<br>29.85<br>No Ct<br>No Ct<br>No Ct<br>(23)	Unknown<br>26.85<br>No Ct<br>38.00<br>No Ct<br>(24)	Unknown<br>29.15<br>30.79<br>No Ct<br>No Ct<br>(25)	Unknown<br>26.69<br>No Ct<br>38.37<br>No Ct<br>(26)	Unknown<br>27.20<br>No Ct<br>No Ct<br>No Ct<br>(27)	Unknown<br>29.31<br>No Ct<br>No Ct<br>No Ct<br>(28)	Unknown<br>29.83<br>No Ct<br>No Ct<br>No Ct<br>(29)	Unknown<br>29.87<br>No Ct<br>No Ct<br>No Ct<br>(30)	
G												
H											NTC<br>No Ct<br>No Ct<br>No Ct<br>No Ct	NTC<br>No Ct<br>No Ct<br>No Ct<br>No Ct

Figure S5: Ct values obtained after qPCR amplification for positive controls, negative controls, patients.
